# A data-driven model for mitochondrial inner membrane remodeling as a driving force of organelle shaping

**DOI:** 10.1242/jcs.263850

**Published:** 2025-06-20

**Authors:** Noga Preminger, Ben Zucker, Sarah Hassdenteufel, Till Stephan, Stefan Jakobs, Michael M. Kozlov, Maya Schuldiner

**Affiliations:** ^1^ Department of Molecular Genetics, Weizmann Institute of Science, Rehovot 7610001, Israel; ^2^ Department of Physiology and Pharmacology, Gray Faculty of Medical & Health Sciences, Tel Aviv University, Tel Aviv-Yafo 6997801, Israel; ^3^ Institute of Molecular Biosciences, Goethe University Frankfurt am Main, Frankfurt am Main 60438, Germany; ^4^ Buchmann Institute for Molecular Life Sciences, Goethe University Frankfurt am Main, Frankfurt am Main 60438, Germany; ^5^ Department of NanoBiophotonics, Max Planck Institute for Multidisciplinary Sciences, Göttingen, Germany; ^6^ Clinic of Neurology, University Medical Center Göttingen, Göttingen, Germany; ^7^ Fraunhofer Institute for Translational Medicine and Pharmacology, Translational Neuroinflammation and Automated Microscopy, Göttingen, Germany

**Keywords:** Mitochondrial shape, Cristae, Membrane remodeling, Organelle shape, Mitochondrial membranes, Biophysical model

## Abstract

Mitochondria are dynamic organelles exhibiting diverse shapes. Although variation in mitochondrial shapes, which range from spheres to elongated tubules, and the transitions between them are clearly seen in many cell types, the molecular mechanisms governing this morphological variability remain poorly understood. Here, we propose a biophysical model for the shape transition between spheres and tubules based on the interplay between the inner and outer mitochondrial membranes. Our model suggests that the difference in surface area, arising from folding of the inner membrane into cristae, correlates with mitochondrial elongation. Analysis of live-cell super-resolution microscopy data supports this correlation, linking elongated shapes to the extent of cristae in the inner membrane. Knocking down cristae-shaping proteins further confirms the impact on mitochondrial shape, demonstrating that defects in cristae formation correlate with mitochondrial sphericity. Our results suggest that the dynamics of the inner mitochondrial membrane are not only important for simply creating surface area required for respiratory capacity but go beyond that to affect the whole organelle morphology. This work explores the biophysical foundations that govern the shape of individual mitochondria, suggesting potential links between mitochondrial structure and function. This should be of profound significance, particularly in the context of disrupted cristae-shaping proteins and their implications in mitochondrial diseases.

## Teaser

**Summary:** A biophysical model demonstrates how cristae formation can drive mitochondrial shape changes, offering a new mechanism linking inner membrane remodeling to organelle shaping beyond fission and fusion.

## INTRODUCTION

Mitochondria are highly dynamic and shape-shifting organelles that display remarkable morphological variations in diverse organisms, cell types, metabolic states, and even within a single cell (Kuznetsov and Margreiter, 2009; Kuznetsov et al., 2006). They can exist both as individual, discrete organelles and as intricate, interconnected networks, continuously shaped by mitochondrial fusion and fission. A single mitochondrion can manifest a wide spectrum of shapes, spanning from a spherical shape through to elongated tubular structures, as well as even more complex shapes such as donuts and lassos (Place et al., 2023; Preminger and Schuldiner, 2024). Spherical mitochondria are evident in pathological states, often seen as mitochondrial swelling, but they also occur in healthy states within specific cell types, like hepatocytes (Das et al., 2012), or within morphologically heterogeneous mitochondrial populations within a single cell (Kuznetsov et al., 2006). In contrast, on the tubular side of mitochondrial shapes, mitochondria can generate lengthy, narrow tubules extending several micrometers within the cell (Rafelski, 2013).

Mitochondria are structurally distinct from other organelles. The lumen of a mitochondrion is enclosed by a double membrane structure consisting of the outer mitochondrial membrane (OMM) and the inner mitochondrial membrane (IMM). The IMM itself can be divided to two distinct subdomains: the inner boundary membrane (IBM), which runs parallel to the OMM, and intricate membrane invaginations known as cristae, which function as the main sites of energy conversion in mitochondria (Cogliati et al., 2016). Cristae, which resemble thin membrane cisternae, extend into the mitochondrial lumen and are connected to the IBM by narrow membrane bridges known as cristae junctions (CJs). The IMM is tightly connected to the OMM by the mitochondrial intermembrane space bridging (MIB) complex (Ott et al., 2012; Tang et al., 2019). The MIB complex is formed by the sorting and assembly machinery (SAM) complex in the OMM and the mitochondrial contact site and cristae organizing system (MICOS) in the IMM. MICOS plays a major role in CJ formation and maintenance (Stephan et al., 2020)*.* The CJs and the cristae are dynamic structures that undergo constant rearrangements and remodeling in response to metabolic and physiological adaptations (Cogliati et al., 2016; Kondadi et al., 2020; Stephan et al., 2019).

Cristae architecture is governed by the interplay of MICOS and several other players, among them the IMM protein optic atrophy 1 (OPA1). OPA1 has a role in mitochondrial fusion and fission as well as in cristae organization and membrane shaping (Anand et al., 2021; Cipolat et al., 2004; MacVicar and Langer, 2016). Depletion of OPA1 leads to a pronounced change in cristae morphology, although it is not essential for the formation of CJs (Barrera et al., 2016). Additionally, the F_1_F_o_-ATP synthase, besides its well-known enzymatic activity as the last step of the respiration pathway, is also a pivotal contributor to cristae structure. The F_1_F_o_-ATP synthase organizes into ribbon-like rows of dimers along cristae ridges and induces high membrane curvature at the rim and tip of cristae (Blum et al., 2019; Davies et al., 2012; Strauss et al., 2008).

Although extensive data have been accumulated on the proteins involved in cristae formation and shaping, the mechanisms responsible for shaping of the OMM, and hence a mitochondrion as a whole, remain largely elusive. Unlike many other tubular structures in the cell – such as the endoplasmic reticulum, T-tubules and intracellular transport intermediates (Kawaguchi and Fujita, 2024; Polishchuk et al., 2009; Wang et al., 2021) – little is currently known about protein-based machinery that could control the shaping of mitochondria and the shift between spherical and tubular shapes independently of fission and fusion (Preminger and Schuldiner, 2024). Notably, even small, discrete mitochondria do not conform to a single shape; they can appear spherical or elongated (Das et al., 2012; Kang et al., 2024; Preminger and Schuldiner, 2024), underscoring that shape can vary at the scale of individual mitochondria and could be determined by factors beyond fission and fusion. Recent years have brought about a barrage of mechanistic studies on proteins important for fission and fusion, and even though these processes explain a considerable portion of mitochondrial shapes and morphological changes, they fall short of fully explaining the diverse array of shapes observed in individual mitochondria. Despite the prevalence of morphological diversity at the level of the single mitochondrion, the molecular and biophysical mechanisms underlying the formation of tubules from spheres, and the regulation of these transitions independently of fission and fusion, remain unknown. This limits our capacity to experimentally control mitochondrial shape in loss/gain-of-function experiments and test the functional significance of mitochondrial structural diversity.

Here, we propose a model for a mechanism for the transition between spherical and elongated shapes of mitochondria, based on the mechanical interplay between the two mitochondrial membranes. We suggest that the driving force for elongation of an individual mitochondrion originates from the area difference between the OMM and the IBM, a difference that arises from the transition of a portion of the IBM area into newly formed cristae, referred to as *de novo* cristae formation. Therefore, our model suggests a correlation between the extent of the mitochondrion shape elongation and the overall area of the cristae. Analysis of live-cell super-resolution data demonstrates a clear relationship between the degree of sphericity of mitochondria and the amount of IMM incorporated into cristae. We further support the model by demonstrating that knockdown of cristae-shaping proteins and disruption of cristae formation lead to observable changes in mitochondrial shapes. These findings propose a new way by which regulation of *de novo* cristae formation can govern mitochondrial shape, with possible implications for aging, neurodegeneration and mitochondrial diseases.

## RESULTS

### Modeling predicts cristae formation as a driving force for organelle elongation

**Fig. 1.**
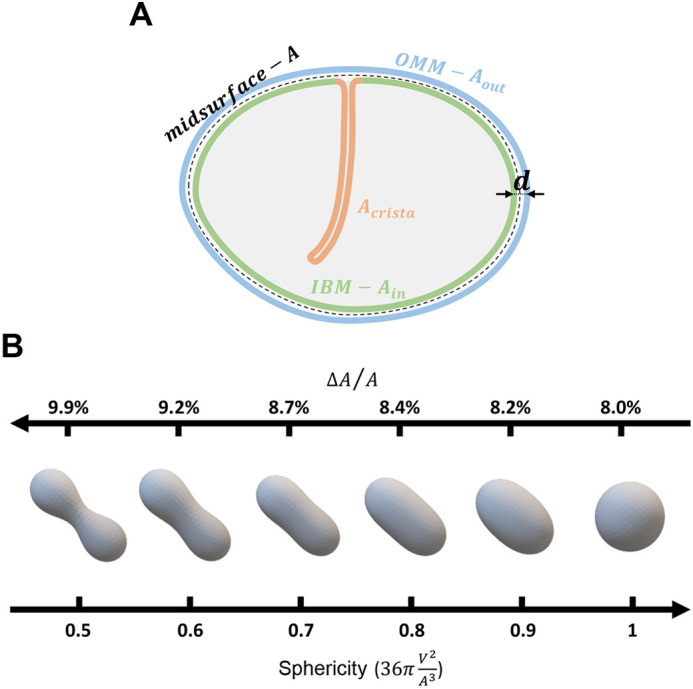
**Modeling membrane surface area as a driving force for organelle shape change.** (A) A schematic representation and main parameters of the model. The OMM is shown by the blue line, and its surface area is denoted by *A*_*out*_. The IMM is composed of (1) the IBM, which is shown by the green curve, and (2) the cristae membrane, which is shown by the orange curve. The surface area of the IBM is denoted by *A*_*in*_. The mid-surface, which is the imaginary surface found between the outer and inner membranes, is shown by the dashed curve, and its surface area is denoted by *A*. The distance between the inner and outer membranes is *d*. (B) Computed results of elastic energy minimization of shapes with a varying difference between the inner and outer membrane surface area. Starting from a spherical shape, as the area difference increases, the sphericity decreases. At small perturbations, the sphere elongates and resembles a prolate ellipsoid. With further increased area difference, the middle part of the shape constricts in a peanut-shaped structure. The normalized area differences, Δ*A*/*A*, of the shapes are calculated for shape evolution starting with a sphere with a diameter of 500 nm and an intermembrane distance, *d*, of 10 nm, while preserving the surface area during the shape evolution.

To support the proposed mechanism of mitochondrial elongation based on the interplay between the mitochondrial membranes, we developed and analyzed a theoretical model. This model considers a system composed of two closed membranes – the outer membrane describes the OMM and the inner membrane represents the IBM. The membranes are parallel to each other with a fixed distance, *d*, between them (Fig. 1A). This distance is maintained by interactions between the two membranes, such as the MICOS-supported contacts between the membranes. Notably, this model is simplistic in that it does not account for the possible addition or removal of lipids to or from the system, which does occur in biological systems. However, our model specifically refers to a short-term period where lipid flow in or out of the membranes is minimal, a scenario that might be more relevant in relatively small mitochondria with limited capacity to form contact sites.

Our model is based on a well-established geometrical principle that governs the shape of a sandwich-like system comprising two closed parallel surfaces. According to this principle, the relationship between the areas of the surfaces dictates the overall shape of the system. Specifically, if the area of the inner surface, *A*_*in*_, is smaller than that of the outer surface, *A*_*out*_, the system tends to adopt an overall elongated (prolate) shape, whereas if *A*_*in*_ increases relative to *A*_*out*_, the shape flattens and becomes oblate and biconcave (Seifert, 1997). This principle underlies the bilayer-couple model of a single membrane, where the two leaflets of the membrane can adopt different surface areas due to variations in their lipid and/or protein content (Svetina and Žekš, 1989). Here, we use this principle to propose that the elongation of a mitochondrion is driven by a decrease in the IBM area, *A*_*in*_, compared to that of the OMM, *A*_*out*_, due to folding of the former into new cristae. We refer to this concept as the CORSET model, which stands for coupled outer–inner membrane rearrangement for shape elongation transition.

To support this proposal quantitatively, we consider the following geometrical and physical arguments (see Materials and Methods for full details). We describe the shape of the system by that of an imaginary mid-surface of area, *A*, which lies between the OMM and IBM parallel to and at a distance *d*/2 from each of them (Fig. 1A). The shape of the mid-surface is determined at each point by the mean curvature, *J*. The overall shape of the mid-surface is characterized by the value of *J* averaged over the surface area, 〈*J*〉. The larger the average curvature, 〈*J*〉, the more elongated and tubular the shape of the system.

The value of the average curvature, 〈*J*〉, is determined by: (1) the difference between the areas of the outer and inner membranes, Δ*A*=*A*_*out*_−*A*_*in*_; (2) the inter-membrane distance, *d*; and (3) the mid-surface area, *A*. A larger ratio Δ*A*/*A* corresponds to a greater average curvature 〈*J*〉, and, hence, a stronger elongation of the shape of the system.

To illustrate the effect of the variations of the area difference, Δ*A*, on the shape of the system, we performed a computation analogous to those of Seifert et al. (1991), whose essence was a minimization of the bending elastic energy of the system and identifying the optimal shapes that minimize it for different values of Δ*A*/*A* (see Materials and Methods). The extent of elongation can be described by simple geometrical measures, such as the sphericity of the shapes, defined as the ratio between the squared volume of the shape and its cubed surface area, normalized by the value of this ratio corresponding to that of a sphere. Hence, a perfect sphere has a sphericity of one. Sphericity decreases as the shape elongates (Fig. 1B). As expected, an increase in Δ*A* drives shape elongation (Fig. 1B). Larger Δ*A*/*A* results in significant elongation and leads to substantial deviations from ellipsoidal shapes towards peanut-like shapes with a slightly constricted middle part (Fig. 1B, left).

### The extent of IMM folded into cristae correlates with tubularity

**Fig. 2.**
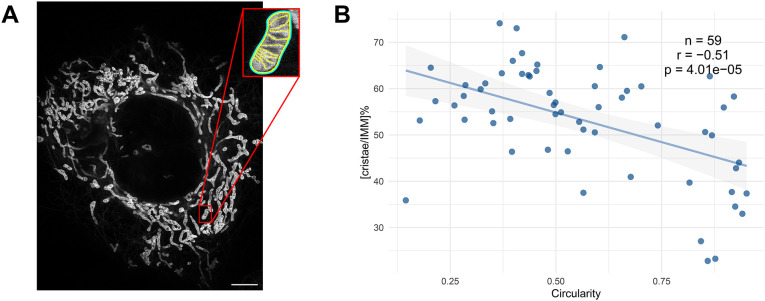
**The extent of cristae correlates with the degree of circularity.** (A) HeLa cells were stained with PKMO (an IMM dye) and imaged using live-cell STED (Liu et al., 2022). The IBM and cristae of individual mitochondria (example in inset) were traced and measured. Scale bar: 5 µm. (B) The percentage of cristae length out of the total IMM length in each mitochondrion, [cristae/IMM]%, was plotted against the circularity measured for the shape of the mitochondrion, where a circularity value of 1 indicates a perfect circle. A Pearson's correlation test (two-tailed) was performed. The line represents a linear regression fit and the shaded area indicates the 95% c.i. *n*, number of analyzed mitochondria; r, Pearson's correlation; p, *P*-value.

The CORSET model predicts that for the initial transition from a spherical mitochondrion to a more tubular shape, more IBM has to be folded into new cristae (or vice versa when cristae unfold back to the IBM). To test this prediction, we assayed whether the shape of mitochondria correlates with the extent of cristae. Since this exact comparison is, in fact, technically challenging, we decided to use, as a proxy, the cristae length as a percentage of the total length of the IMM (which includes both the IBM and the cristae), herein referred to as [cristae/IMM]%. To do this, we analyzed images of mitochondria from live HeLa cells acquired by two-dimensional (2D) live-cell stimulated emission depletion (STED) microscopy, in which the entire IMM was stained with the mitochondrial dye PK Mito Orange (PKMO) (Liu et al., 2022). Mitochondrial shape was traced and assessed for its sphericity (Fig. 2A). To determine sphericity, we calculated the 2D circularity, defined as 4*π*(*area*/*perimeter*^2^). Indeed, when [cristae/IMM]% was calculated, we observed a correlation between circularity and the percentage of IMM incorporated into cristae, indicating that more spherical mitochondria had lower [cristae/IMM]% (Fig. 2B). A similar trend was observed when the calculations were transformed from 2D to an inferred three-dimensional (3D) prediction, considering the surface area of disk-shaped cristae and the surface area of the cylinder-shaped boundary membrane (see Fig. S1A,B).

Our model is relevant only to the primary shape change – from a sphere to an elongated (‘bean-like’) shape, but does not account for further extension of larger, longer tubules. We therefore examined the relationship between shape circularity and [cristae/IMM]% among different size groups of mitochondria, which could indicate whether they are in the relevant initial shaping stage or have already progressed beyond it. To discern distinct shaping behaviors, the pool of all mitochondria was divided based on the area of the mitochondrion (measured as the 2D area found inside the perimeter of the IBM), using the median value to create two groups (designated as ‘small’ for the ones found below the median and ‘large’ for those above it, based on their respective areas) (Fig. S2A). Notably, there was no significant difference in diameter between the two groups (data not shown), indicating that increased area mainly stemmed from difference in length of the mitochondrion. The calculated [cristae/IMM]% was then plotted against the circularity (Fig. S2B). Indeed, different trends were observed in the small- and large-sized mitochondria groups: whereas the small-sized mitochondria showed a significant negative correlation between [cristae/IMM]% and circularity, as expected according to the proposed model, no significant correlation was observed in larger mitochondria. These results support a biphasic behavior of the mitochondrial shaping process: an initial shape transition governed by *de novo* cristae formation, as suggested by the model, followed by further shape extension, likely by fusion of mitochondria and external forces exerted by various interactions with cellular components. During this extension into longer tubules, cristae likely continue to form in order to maintain the IMM surface area required for respiration, rather than to influence organelle shape. Therefore, we posit that shaping by the interplay of IMM–OMM area difference is relevant only to the transition from a perfect sphere to a more elongated shape at earlier stages of growth of the mitochondrion, a process that is independent of addition of lipids, but cannot explain the following extensive elongation of mitochondria into long narrow tubules, a stage that requires membrane expansion and increase in lipid mass through fusion or lipid transfer via contact sites.

### Knockdown of cristae-shaping proteins affects mitochondrial shape

**Fig. 3.**
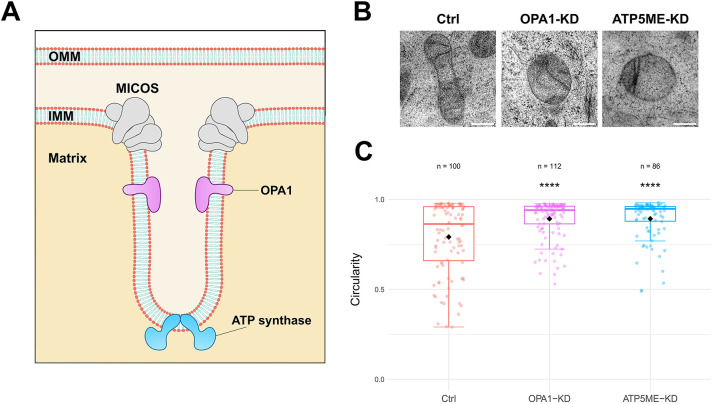
**Knockdowns of cristae-shaping proteins affect mitochondrial shape.** (A) Scheme of a crista and cristae-shaping proteins. (B) Representative EM recordings of mitochondria in HeLa cells transfected with a control (Ctrl) siRNA pool or an siRNA pool for knockdown (KD) of cristae-shaping proteins, as indicated. Scale bars: 200 nm. (C) Circularity measurements of mitochondria sampled from Ctrl and KD cells. Each dot represents one mitochondrion. Horizontal lines within boxes indicate the median, black dots indicate the mean. Boxes show the interquartile range (IQR), and whiskers extend to 1.5× IQR from the Q1 and Q3 boundaries (Tukey method). Student's t-test (unpaired two-tailed) was used to compare to the control. *n*, number of analyzed mitochondria; ****P≤0.0001.

If indeed the formation and maintenance of cristae has an impact on the transition from a spherical to elongated shape of the mitochondrion, then changes in the proteins involved in these processes should impact the sphericity of mitochondria in cells. To test this, we used small interfering RNA (siRNA) to knock down central proteins involved in cristae formation and maintenance. The MICOS complex, besides its central role in IMM shaping and cristae formation, is also involved in the formation of the MIB complex, which connects the OMM and IMM (Kozjak-Pavlovic, 2017; Ott et al., 2012). Since the integrity of these membrane contact sites is crucial for our model, we did not use MICOS knockdowns that might disrupt these connections and focused instead on other proteins involved in cristae shaping – OPA1 and the F_1_F_o_-ATP synthase subunit e ATP5ME (Fig. 3A; Fig. S3). Electron microscopy (EM) analysis confirmed that cristae architecture was altered when these proteins were knocked down, resulting in several different cristae morphologies (Fig. 3B). We assessed the shape of individual mitochondria in these cells, comparing them to mitochondria from control cells. Mean circularity of mitochondria was significantly different between control and knockdown cells (Fig. 3C). It is important to note that although OPA1 and the ATP synthase also affect mitochondrial fission and fusion (Cipolat et al., 2004; Lhuissier et al., 2024), our analysis focused on individual, discrete mitochondria. Even within this isolated or fissioned state, mitochondria can still adopt either spherical or elongated shapes independently of fusion capability. Knockdowns of OPA1 and ATP5ME resulted in a statistically significant shift in the shape of mitochondria to a more spherical shape, as would be predicted by the CORSET model, suggesting that indeed, reduced capacity to form cristae results in less capacity to elongate.

### Time-resolved shape transitions support a correlation between shape and cristae extent

**Fig. 4.**
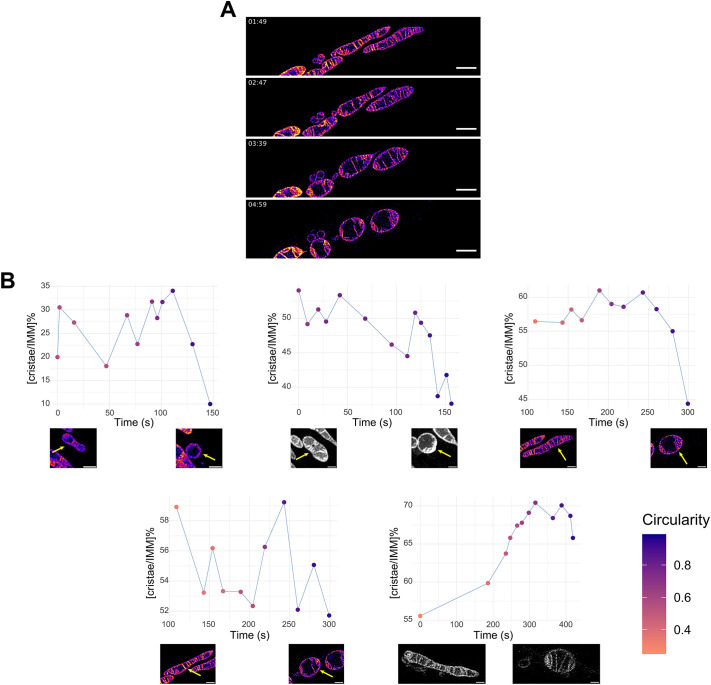
**Time-resolved analysis of cristae extent during mitochondrial shape transition.** (A) Representative snapshots extracted from time-lapse STED recordings (taken from Wang et al., 2019; republished with permission), showcasing the morphological transformation of mitochondria from tubular to spherical shapes over time (min:sec). Mitochondria were stained using MitoPB Yellow, enabling visualization of the IMM (Wang et al., 2019). MitoPB Yellow is shown in a false-color scale (magenta to yellow) representing fluorescence intensity. Scale bars: 2 µm. (B) Graphs depicting the change in [cristae/IMM]% over time (seconds) for mitochondria undergoing the transformation exemplified in A. Each graph represents one individual mitochondrion (shown in the pictures below each graph for initial and final times tracked, arrows indicating the mitochondrion analyzed), with color coding of the graphs indicating the degree of circularity of the mitochondrial shape at each timepoint. MitoPB Yellow is shown in a magenta–yellow scale and grayscale. Scale bars: 1 µm. Images taken from Wang et al., 2019. (republished with permission).

Examination of still images allowed us to observe a correlation between the overall shape of mitochondria and the proportion of IMM folded into cristae. While static images provide information about the relationship between shape and cristae folding at a specific point, they only offer a snapshot of a dynamic, continuously evolving process. To delve deeper into this interplay, we conducted a time-resolved analysis using previously published time-lapse STED recordings of mitochondria stained with MitoPB Yellow (Wang et al., 2019), imaged over prolonged periods. It was shown in this previous study that during long-term observations (>90 s) mitochondria undergo a drastic change in their shape, due to photodamage induced by the intense STED lasers, resulting in a progressive loss of cristae (Wang et al., 2019). During this process, cristae are observed to gradually retract into the IBM, and within minutes the mitochondria transition from narrow tubules to complete spheres (Fig. 4A). Importantly, these shape transitions occur without any fission events. While the precise processes driving these changes are not entirely clear and other processes in these mitochondria might be at play (Icha et al., 2017; Knight et al., 2003; Magidson and Khodjakov, 2013), the observation that cristae retraction correlates with increased mitochondrial sphericity provides insights into how cristae remodeling might influence overall mitochondrial shape. Already this initial description aligns with our model – reduced cristae and increased IBM preceding a change in shape to spherical mitochondria. To validate this, we assessed the mitochondrial shape (circularity) and the extent of cristae, quantified as [cristae/IMM]%, at several timepoints during the shape change process. Despite some fluctuations (which might be a by-product of the dynamic cristae moving in and out of the focal plane, thereby somewhat affecting the measurements), an overall reduction in [cristae/IMM]% was observed from the initial tubular shape to the final spherical shape in the majority of mitochondria analyzed (Fig. 4B). Although the photodamage could induce various changes in mitochondria and consequently in their shape, the association between higher sphericity and a reduction in cristae extent supports our proposed model and highlights the relevance of cristae formation in the shaping of the whole organelle. Analysis of the time-resolved shift in live mitochondria further suggests that the folding or unfolding of cristae can serve as a mechanism to alter the surface area of the IBM, potentially contributing to the complex process of mitochondrial shape transition.

## DISCUSSION

Research of mitochondrial shape has so far been dominated by exploration of the network formation, with a clear focus on the fission and fusion processes that are fundamental in shaping it. Clearly, fission and fusion also affect the length of an individual mitochondrion. However, they cannot fully explain every transition of mitochondria from a sphere to elongated shapes.

**Fig. 5.**
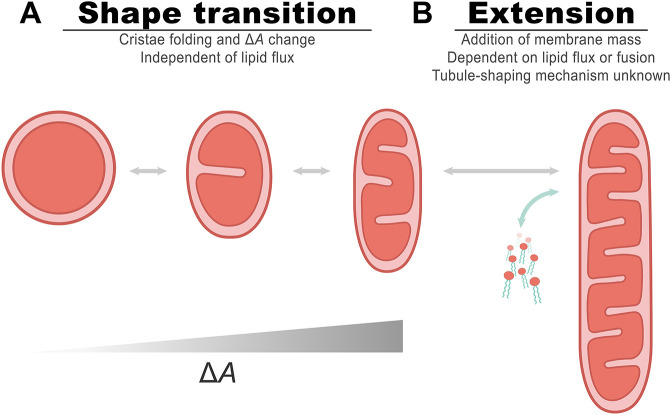
**The CORSET model for shaping mitochondria.** The shift of mitochondria from spheres to tubules or vice versa can be divided into two substages. (A) The first step is the change from a sphere to a primary tubule (the classical bean-like shape), which is hypothesized to be driven by cristae formation and changes in area difference (Δ*A*) between the membranes. (B) The second step is a further extension to a full-length tubule by either growth or fusion of several mitochondria, meaning an external addition of lipids, to create longer narrow tubules.

In this study, we explored the possible biophysical mechanisms that govern this initial basic stage of mitochondrion shaping. The process of *de novo* cristae formation clearly affects the ultrastructure of the IMM and therefore the surface area supporting the electron transfer chain machinery and consequently cellular respiration. Yet, we suggest that it is also involved in regulating the entire organelle shape, thereby introducing an additional layer of complexity to cristae functionality. Folding of the IMM into cristae results in a reduction of the surface area of the IBM, and thus, increases the difference between the surface area of the IBM and OMM. Based on geometrical considerations and computations of membrane shapes, we propose that the resulting change in the surface area difference, coupled to the strong physical connectivity between the OMM and IBM, can drive a small, isolated mitochondrion to transition from a sphere to an elongated form (Fig. 5A). This implies an interplay between the two membranes, where the OMM follows the action of the IMM, contrary to known processes like fission and fusion, where changes typically initiate with the OMM and progress to the IMM. Although current methodologies make it challenging to definitively prove this model experimentally, analyses of mitochondrial shapes in live cells support the possibility of this hypothesis, as we observed that spherical mitochondria tended to have less of their IMM packed into cristae, relative to more tubular mitochondria in which a larger portion of the IMM was observed in cristae. This was evident in both static images and time-resolved recordings during active shape changes.

Attempts to validate this theoretical model face various biological and technical constraints. First, the model describes a dynamic membrane remodeling process that occurs in the subdiffraction resolution realm and is therefore challenging to track over time with sufficient resolution. Second, the reliance on 2D measurements to infer 3D shapes introduces inherent limitations in shape descriptors. While our current analysis is based on 2D projections, we acknowledge the limitations this imposes on interpreting 3D membrane geometry. Although 3D tomographic reconstructions could help refine the geometrical assumptions of our model, such datasets remain scarce and difficult to obtain at sufficient throughput for quantitative analysis. Consequently, we opted for two complementary methods: 2D super-resolution microscopy and EM. EM provides outstanding resolution but requires thinner slices. This makes cristae, which are small relative to the overall organelle, easy to miss if the slice position shifts even slightly. Such shifts can result in inaccurate measurements of cristae extent. Thus, to minimize the possibility of such artifacts, we analyzed cristae extent in super-resolution images of live cells (Jakobs et al., 2020) and used EM for shape measurements of the whole organelle.

Our model describes a shape change that does not require, and even precludes, the addition of lipids to either of the membranes. This assumption is crucial to explain the folding of cristae as a driver of area difference between the membranes. Of course, this also makes it extremely challenging to test in a cell, where contact sites are constantly contributing to new lipid uptake by mitochondria (Sassano et al., 2022; Scorrano et al., 2019). Indeed, in live dynamic mitochondria, lipid addition and loss are constantly occurring, and thus it is important to emphasize that our model is reductionistic in its nature and does not exclude the influence of other processes known to affect membrane and organelle shape. This model does not dismiss lipid metabolism or lipid-based models that have recently been proposed (Venkatraman et al., 2023), which highlight the mechanical role of lipid composition, especially cardiolipin, in generating curvature and stabilizing cristae structure. Instead, we propose that, under conditions with minimal lipid movement, membrane proteins like MICOS, OPA1 and ATP synthase oligomers support cristae formation by providing the necessary mechanical forces and membrane curvature for cristae shaping. The mechanism we propose applies for specific, short-term cases – for example where the mitochondrial shape has to change without affecting the lipid content or in the absence of functional lipid uptake – and is clearly only one of many complementary processes and mechanisms that help control the shape of mitochondria.

The CORSET model underscores the possible role of *de novo* cristae formation in generation of area difference between the membranes, but it may also involve additional parameters that contribute to shape regulation. For instance, the consistent inter-membrane distance maintained by contact sites is an essential factor for the feasibility of this model. Disruption of this factor could potentially impede the shaping process, serving as another avenue for regulation of this mechanism besides the cristae-shaping machinery. Thus, it is of future interest how changes or regulation of all parameters considered in the model affect whole mitochondrial morphology.

Although we focused our analysis on one biological system, HeLa cells, for consistency, compatibility with imaging techniques and robustness of shape quantification, the core biophysical mechanism described by the CORSET model is expected to apply broadly across eukaryotes. Future work could explore its relevance in a wider range of organisms with diverse mitochondrial architectures, such as the budding yeast, where cristae remodeling has been extensively studied and where additional mutant cristae morphologies have been observed (Harner et al., 2016; Klecker and Westermann, 2021).

Analysis of mitochondrial shapes in live cells revealed that the suggested mechanism can only explain the shapes of a certain population of mitochondria that is defined by their small size. This, together with the underlying assumption of the model that there is no lipid addition, emphasizes two key points. Firstly, the applicability of this model is primarily to smaller, isolated mitochondria rather than to elongated tubules or those within the mitochondrial network. Secondly, it suggests a biphasic nature of shape change in mitochondria. The overall shift between a sphere to a long narrow tubule can be divided into two. The first step is the transition from a sphere to a primary tubule (the classical bean-like shape) (Fig. 5A), which could be driven mostly or completely by *de novo* cristae formation and change in area difference between the membranes, as explained by our model. Importantly, it is not the mere presence of cristae and CJs that helps stabilize the tubules, but rather the folding of new cristae that can drive this shape change, due to the decrease in the surface area of the IBM. The second step of the shape shift is the subsequent extension to a full-length tubule by growth or fusion of several mitochondria to create longer tubules (Fig. 5B). Elongation at this second step cannot be explained by cristae formation alone, as it is constrained by the lower limit of width of an individual mitochondrion. This lower limit of width represents a physical constraint that impedes additional elongation solely through cristae formation. Essentially, there is a point at which the mitochondrion cannot narrow any further without compromising its structural stability or functional efficiency. In addition, at a certain point expansion of the membranes will require, and even depend on, supplementation of lipids or fusion of entire mitochondrial units. Beyond representation of a substage towards longer tubules, the first step in our model might also allow for a fast and/or transient short-term shift in shape, without the profound changes and global involvement associated with lipid uptake. Hence, it is essential to distinguish between the substages of shape transition and elucidate the mechanisms, forces, kinetics and components required for each one.

Spherical mitochondria are observed in various conditions and cell types, both in healthy and pathological states. For example, the spherical mitochondria seen in hepatocytes (Das et al., 2012) or the mitochondria in skeletal muscles, which range from small round mitochondria close to the sarcolemma to long tubular mitochondria located at the core of myofibers (Picard et al., 2013). While the majority of focus in the literature has been on the pathological state, often referred to as ‘swelling’, our results suggest that sphericity should be regarded as a normal part of the mitochondrial shape repertoire. It remains unclear whether spherical shapes are just a step on the way to further elongation to a tubule or whether they hold some functional significance. The biphasic model might suggest that mitochondrial fusion is only possible after cristae-induced mitochondrial elongation. To study questions regarding the functional importance of spherical and tubular shapes, we must be able to control this shape transition experimentally. However, the regulation that governs the decision on mitochondrial shape and the transition between one shape to another is an unexplored area that requires further research.

The link between mitochondrial diseases and malfunction of cristae-shaping proteins has been clearly demonstrated, with detrimental effects observed at the organellar, cellular and organismal level (Cogliati et al., 2016; Colina-Tenorio et al., 2020). The impact of disrupted cristae shaping and remodeling has been attributed mainly to dysfunction of respiratory processes, which occur in the cristae and are greatly impacted by their shape. We suggest taking into consideration the effect of impaired cristae formation on the overall shape of mitochondria, and exploring the direct implications that this could have for mitochondrial and cellular function beyond the clear impact on energy conversion.

## MATERIALS AND METHODS

### Theoretical modeling and analysis

#### Geometrical formulation

To describe the geometry of the system, we considered three mutually parallel surfaces. The outer and inner membranes are represented by their mid-surfaces lying between the membrane leaflets and having the areas *A*_*out*_ and *A*_*in*_, respectively (Fig. 1A). The system as a whole is described by a surface, referred below to as the system surface, that is parallel to and lies at half the distance between the surfaces of the outer and inner membranes (Fig. 1A). The area of this mid-surface is denoted by *A*.

The local shape of the system surface at each point is described by two principal curvatures, *c*_*p*1_ and *c*_*p*2_, which are the curvatures of two arc-like cross-sections of the surface by normal planes in the specific directions called the principal directions (Spivak, 1970). The sign of each principal curvature is conventionally defined according to the convexness (positive sign) or concaveness (negative sign) of the corresponding cross-section line. Alternatively, the local surface shape can be described by the sum and product of the principal curvatures, *J*=*c*_*p*1_+*c*_*p*2_ and *K*=*c*_*p*1_ · *c*_*p*2_ (Helfrich, 1973), referred to as the total and Gaussian curvature, respectively. In the following, we will assume that the absolute values of the curvatures, |*c*_*p*1_| and |*c*_*p*2_|, and hence |*J*|, are small all along the system surface compared to the inverse distance between the outer and inner membranes, 1/*d*, such that their dimensionless combinations are smaller than one: |*c*_*p*1_|*d*≪1, |*c*_*p*2_|*d*≪1 and |*J*|*d*≪1. In this case, the dimensionless value of the Gaussian curvature is smaller than that of the total curvature, |*K*|*d*^2^≪|*J*|*d*, so that in all expressions below we will neglect the contributions related to *K*. For brevity, we will refer to *J* as simply the curvature.

Since all three surfaces are mutually parallel, their local geometrical characteristics are interrelated. The area elements, *dA*_*out*_ and *dA*_*in*_, of the outer and inner membrane surfaces, respectively, are related to the element of the system surface, *dA*, by the relationships (see, for example, Murphy, 1966):
(1)

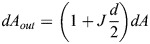
and
(2)

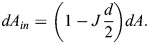


These expressions (Eqns 1,2) enable determination of a qualitative relationship between the overall character of the system shape and the difference between the outer and inner membrane areas, *A*_*out*_ and *A*_*in*_. Indeed, the outer and inner membrane areas are given by the integration of the area elements, 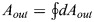 and 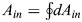, so that, according to Eqns 1 and 2, they can be expressed by:
(3)

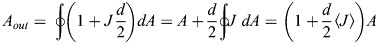


and
(4)

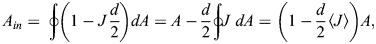
where 〈*J*〉 is the curvature of the system surface averaged over the surface area, *A*. Combining Eqns 3 and 4, we obtain:
(5)

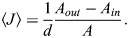


According to Eqn 5, the larger the difference between the outer and inner membrane areas, *A*_*out*_−*A*_*in*_, the larger the average curvature of the system surface 〈*J*〉.

#### Elasticity analysis

The areas of the outer and inner membranes, *A*_*out*_ and *A*_*in*_, respectively, determine the average curvature, 〈*J*〉, but do not set the exact shape of the system surface.

To find the specific shape of the system surface, we consider the elastic energy of the system, *F*. The optimal configuration of the system is determined by minimization of the sum of the elastic energies of the outer (*F*_*out*_) and inner (*F*_*in*_) membranes, *F*=*F*_*out*_+*F*_*in*_, by varying the system shape within the constraints imposed by the given areas *A*_*out*_ and *A*_*in*_ and the given distance, *d*, between the outer and inner membrane surfaces.

We assume the only contribution to the elastic energy of the membranes to be the bending energy, which implies that the membranes are considered to be non-stretchable. To compute the elastic energies of the outer and inner membranes we use the Helfrich model (Helfrich, 1973), according to which the bending energy of a membrane element related to the membrane unit area depends on the membrane total curvature and can be represented by:
(6)

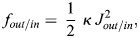
where *κ* is the bending modulus (Helfrich, 1973) and the subscript indicates which membrane is described. The curvature of each membrane could be inferred from the middle surface by geometrical considerations (Murphy, 1966):
(7)

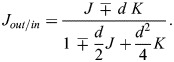


Since, according to Eqn 7, the deviations of *J*_*out*/*in*_ from *J* are quadratic in the product of the principal curvatures and the membrane thickness, they will be neglected.

The total elastic energy, *F*_*in*/*out*_, is obtained by integrating *f*_*in*/*out*_ over the area of the corresponding membrane surface:
(8)

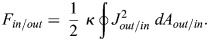


By using Eqn 6 we assume the membranes to have a vanishing spontaneous curvature (Helfrich, 1973), which implies that for each membrane the two leaflets are similar so that the membrane structure is up–down symmetric. Such computation of the optimal configuration of our system is technically analogous to the previous analysis of the shapes of individual membranes determined by the bilayer-couple model (Seifert, 1997; Svetina and Žekš, 1989).

We numerically performed the minimization of the system bending energy (Eqn 8) upon a constraint of constant 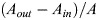 and found the optimal shapes of the system surface by using the specialized program Brakke's Surface Evolver (Brakke, 1992). The results for a few values of the relative difference between the outer and inner membrane areas, 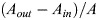, are presented in (Fig. 1B). As predicted by the geometrical analysis, an increase in 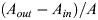 drives the shape elongation. Indeed, the obtained optimal shapes resemble ellipsoids when the deviation from a sphere is small. However, for significant elongation, the shapes deviate substantially from ellipsoids, acquiring a peanut-like shape with a slightly constricted middle part.

### Cell culture for live-cell imaging

HeLa cells (ATCC CCL-2) were grown in Dulbecco's Modified Eagle Medium (DMEM) with GlutaMAX additive and 4.5 g/l glucose (Thermo Fisher Scientific, USA). The culture medium was supplemented with 1% (v/v) penicillin-streptomycin (Sigma Aldrich, Munich, Germany), 1 mM sodium pyruvate (Sigma Aldrich) and 10% (v/v) fetal bovine serum (FBS; Merck Millipore, Burlington, MA, USA). Cells were cultured in an incubator at 37°C with 5% CO_2_. Cells were frequently tested for mycoplasma contamination by PCR.

### PKMO labeling for live-cell imaging

HeLa cells were stained as described previously (Liu et al., 2022). In brief, HeLa cells were seeded in glass-bottom dishes (ibidi GmbH, Germany) 1 day prior to imaging. Cells were stained with DMEM containing 350 nM PKMO (Liu et al., 2022), 200 nM 4-610CP-CTX (tubulin dye, not shown; Bucevičius et al., 2020), and 0.2 μl/ml Quant-iT PicoGreen reagent (mtDNA dye, not shown; Thermo Fisher Scientific) at 37°C for 40 min. Following the staining procedure, cells were washed three times with culture medium and incubated at 37°C for 60 min to remove unbound dye. The cells were imaged in DMEM buffered by 4-(2-hydroxyethyl)-1-piperazineethanesulfonic acid (HEPES) at room temperature.

### Live-cell imaging

HeLa cells were recorded using an Expert Line dual-color STED 775 QUAD scanning microscope (Abberior Instruments) equipped with a UPlanSApo 100×/1.40 Oil [infinity]/0.17/FN26.5 objective (Olympus). PKMO was excited at 561 nm wavelength, and STED was performed using a pulsed depletion laser at 775 nm wavelength with gating of 1–7 ns and dwell times of 5–10 μs. Pixel sizes of 25–28 nm were used for STED nanoscopy and each line was scanned 3–10 times (line accumulations). The pinhole was set to 0.7–1.0 AU.

### Image analysis for STED microscopy

Images of PKMO-labeled cells were visualized and analyzed using Fiji (version 1.54f; Schindelin et al., 2012). For the analysis, non-branched linear mitochondria with well-labeled cristae were chosen (only when a clear and continuous boundary could be visualized); mitochondria that did not have any labeled cristae were excluded from the analysis (to this end, our measurements may be an underestimate to the degree of correlation since we do not have the ‘no cristae’ population, which may definitely exist in cells). The boundary shape of mitochondria and individual cristae were manually traced using the freehand selection tool and the selection brush tool in Fiji, and then measured for their shape descriptors. The measured length of each crista was doubled to account for the fact that each crista consists of two membrane layers. The sphericity of mitochondrial shapes was evaluated by the 2D circularity: 4*π*(*area*/*perimeter*^2^). For validation of measurement accuracy, we ensured that that our values for several measurements (mitochondrion area, perimeter and diameter) matched those commonly reported in the literature (Chu et al., 2022; Wiemerslage and Lee, 2016; Yang et al., 2020).

#### Three-dimensional transformation of shape measurements

To assess the 3D measurements of analyzed mitochondria from STED images, we estimated the shape of a mitochondrion as a cylinder with hemispherical ends, with a radius of *R* (Fig. S1A). To assess the 3D surface area of the boundary membrane (*S*_boundary_) of a mitochondrion, we first considered a ring-shaped portion of the surface area, with a radius of *R* and a width of *L*_1_ (Fig. S1A). The surface area of such shape will be:

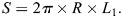


Extending this to the whole boundary membrane (which consists of many such ring-shaped portions), the surface area for each mitochondrion was approximated as:

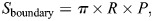
where *P* is the perimeter measured by 2D tracing of mitochondria, and *R* is half of the diameter manually measured for each mitochondrion.

To estimate the surface area of cristae, cristae were considered as flat disks with a diameter of *L*_2_ (Fig. S1A). Hence, the surface area of a crista is:

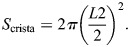


The surface area of all cristae was calculated in all analyzed mitochondria, where the length measured of each traced crista was used as *L*_2_.

The [cristae/IMM]% of each mitochondrion was calculated by:

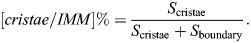


### Cell culture and transient knockdowns for EM

HeLa cells (ATCC CCL-2) were cultivated at 37°C in DMEM with GlutaMAX additive, 4.5 g/l glucose and 1 mM sodium pyruvate (Thermo Fisher Scientific, USA), supplemented with 10% FBS (Thermo Fisher Scientific, USA) and 1% penicillin-streptomycin (Biological Industries, Israel) in a humidified environment with a 5% CO_2_ atmosphere. Cells were frequently tested for mycoplasma contamination by PCR. For 72 h knockdown of OPA1 or ATP5ME, 2×10^5^ HeLa cells were seeded into a 60 mm culture dish with regular culture medium. Cells were transfected with a high-complexity siRNA pool targeting OPA1 (NCBI Gene ID 521) or ATP5ME (NCBI Gene ID 4976) (both synthesized by siTOOLs Biotech, Germany) to a final concentration of 3 nM using Lipofectamine RNAiMAX transfection reagent (Thermo Fisher Scientific, USA) according to the manufacturer's instructions. All knockdowns were verified by western blotting. Specifically, cells were lysed in RIPA buffer (Sigma-Aldrich) supplemented with a protease inhibitor cocktail (Calbiochem) and protein concentrations were determined using the BCA assay (Pierce). Equal amounts of protein (20–30 µg) were separated by SDS-PAGE on Tris-Glycine polyacrylamide gels (12% or 19% with Urea) and transferred to nitrocellulose membranes (Pall corporation) using a semi-dry transfer system (Bio-Rad). Membranes were blocked in 3% non-fat dry milk in PBS for 1 h at room temperature and incubated overnight at 4°C with primary antibodies diluted in blocking buffer. The following antibodies were used: rabbit anti-OPA1 (1:1000; 67589, D7C1A, Cell Signaling Technology, USA]), rabbit anti-ATPase subunit e (1:1000, 16483-1-AP, Proteintech, USA) and mouse anti-β-actin (1:2000; Abcam, 8224) as a loading control. After washing, membranes were incubated with fluorescently labeled secondary antibodies: IRDye 800CW goat anti-rabbit IgG (1:10,000, 925-32211, Li-COR, USA); IRDye 680RD goat anti-rabbit IgG (1:15,000, 26-68070, Li-COR, USA) for 1 h at room temperature. Fluorescentce signals were detected using the Odyssey Imaging System (LI-COR).

### EM sample preparation and imaging

EM was performed following a standard sample preparation (Kukulski et al., 2012; Bykov et al., 2019). In short, a viscous cell suspension was transferred to the 0.1 mm-deep cavity of a 0.1/0.2 mm membrane carrier for a Leica ICE high-pressure freezing machine. The cavity was covered by the flat side of a 0.3 mm carrier, and the sandwich was inserted in the high-pressure freezing machine. Resin embedding was performed using a Leica AFS2 freeze substitution machine equipped with a processing robot. Samples were embedded in Lowicryl HM20 resin using the freeze substitution and embedding protocol optimized for in-resin CLEM (Kukulski et al., 2012). Dry acetone with 0.1% uranyl acetate was used as the freeze substitution medium. The blocks were trimmed using a Diatome 45° trimming knife, and 100 nm-thick sections were produced using a Diatome 35° knife on a Leica UC7 microtome. The sections were mounted on 200 mesh copper grids with continuous carbon support film (Electron Microscopy Sciences). Prior to EM, grids were triple post-stained: 1 min in Reynolds lead citrate, 10 min in uranyl acetate and 1 min in Reynolds lead citrate. EM imaging was performed on a FEI Tecnai G2 F20 TEM microscope operating at 120 kV, equipped with a TVIPS TemCam-XF416 retractable 16 megapixel CMOS camera. SerialEM software was used for data collection (Mastronarde, 2005; Schorb et al., 2019), and the detector was operated in the full-frame mode (4096×4096 pixels). To cover a complete cell, a 3×3 montage was acquired with a magnification of 6500× (corresponding to a pixel size of ∼1 nm).

### Image analysis for EM

EM data were visualized and analyzed using Fiji (version 1.54f; Schindelin et al., 2012). The median filter in Fiji was applied to images to reduce noise. Usually, 80–110 mitochondria from at least eight different cells were randomly selected and analyzed for each sample. The boundary shape of mitochondria was manually traced using the freehand selection tool and the selection brush tool in Fiji, and then measured for its shape descriptors. The sphericity of mitochondrial shapes was evaluated by the 2D circularity: 4*π*(*area*/*perimeter*^2^).

## Supplementary Material

10.1242/joces.263850_sup1 Supplementary information
