# Supplementary material for: A data-driven model for mitochondrial inner membrane remodeling as a driving force of organelle shaping
Source: J Cell Sci. 2025 Jun 20;138(12):jcs263850. doi: 10.1242/jcs.263850 (PMC12211560; doi:10.1242/jcs.263850)
Supplement: Supplementary information [file joces-138-263850-s1.pdf]

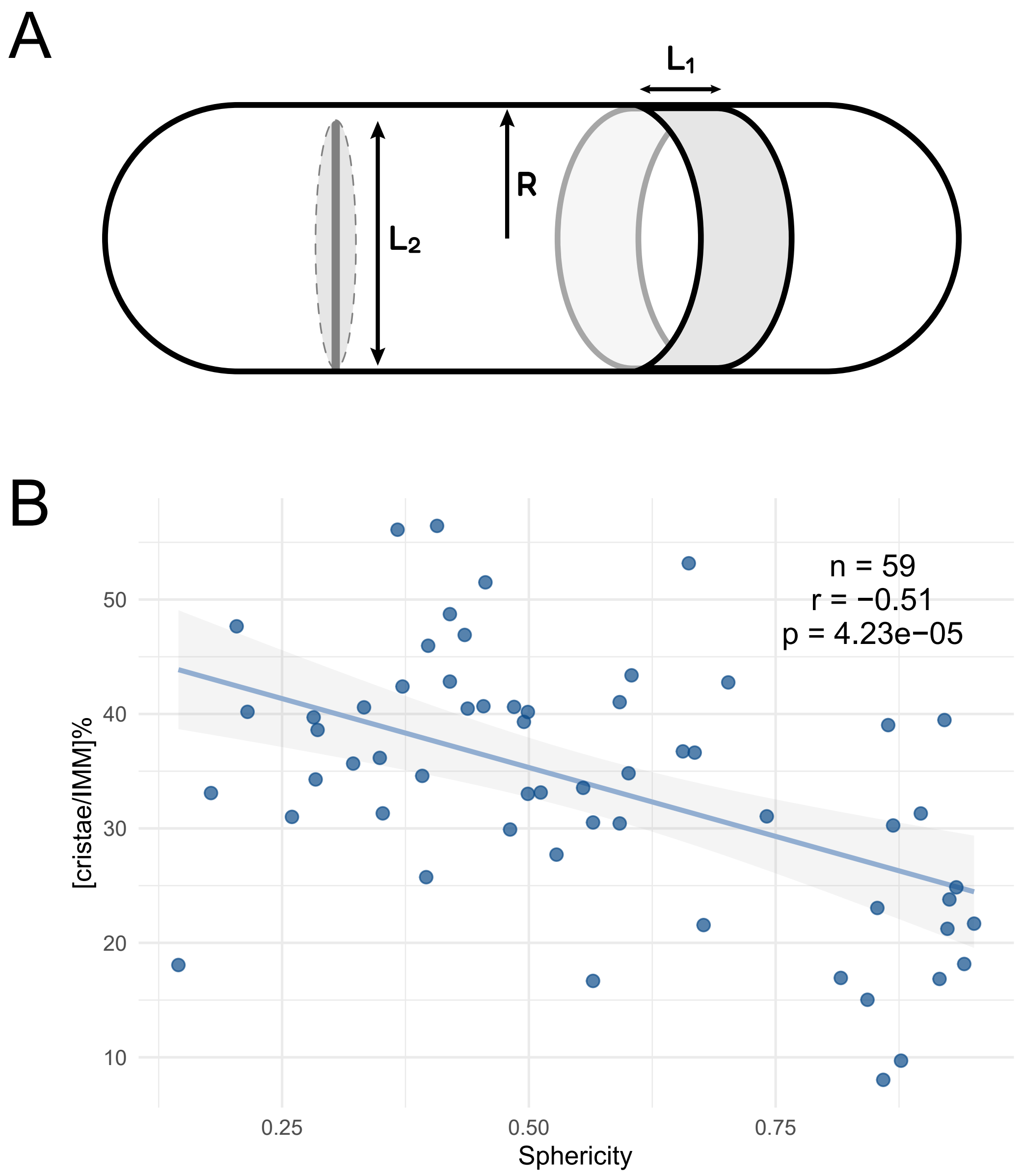

Fig. S1. Transformation from two-dimensional (2D) to three-dimensional (3D) calculation maintains a negative correlation between cristae extent and sphericity. (A) A scheme of a mitochondrion in the shape of a cylinder with hemispherical ends, with a radius R. In grey, a ring-shaped portion of the boundary surface area with the width L_1, and a disk-shaped crista with a diameter of L_2. For details of 2D-to-3D transformation see Materials and Methods. (B) After estimation of 3D measurements, the percentage of cristae surface area out of the total surface area of the IMM in each mitochondrion ([cristae/IMM]%) was plotted against the sphericity measured for the shape of the mitochondrion, where a sphericity value of 1 indicates a perfect sphere. A Pearson's correlation test (two-tailed) was performed. The line represents a linear regression fit and the shaded area indicates the 95% confidence interval. n, number of analyzed mitochondria; r, Pearson's correlation; p, P-value.

Journal of Cell Science • Supplementary information

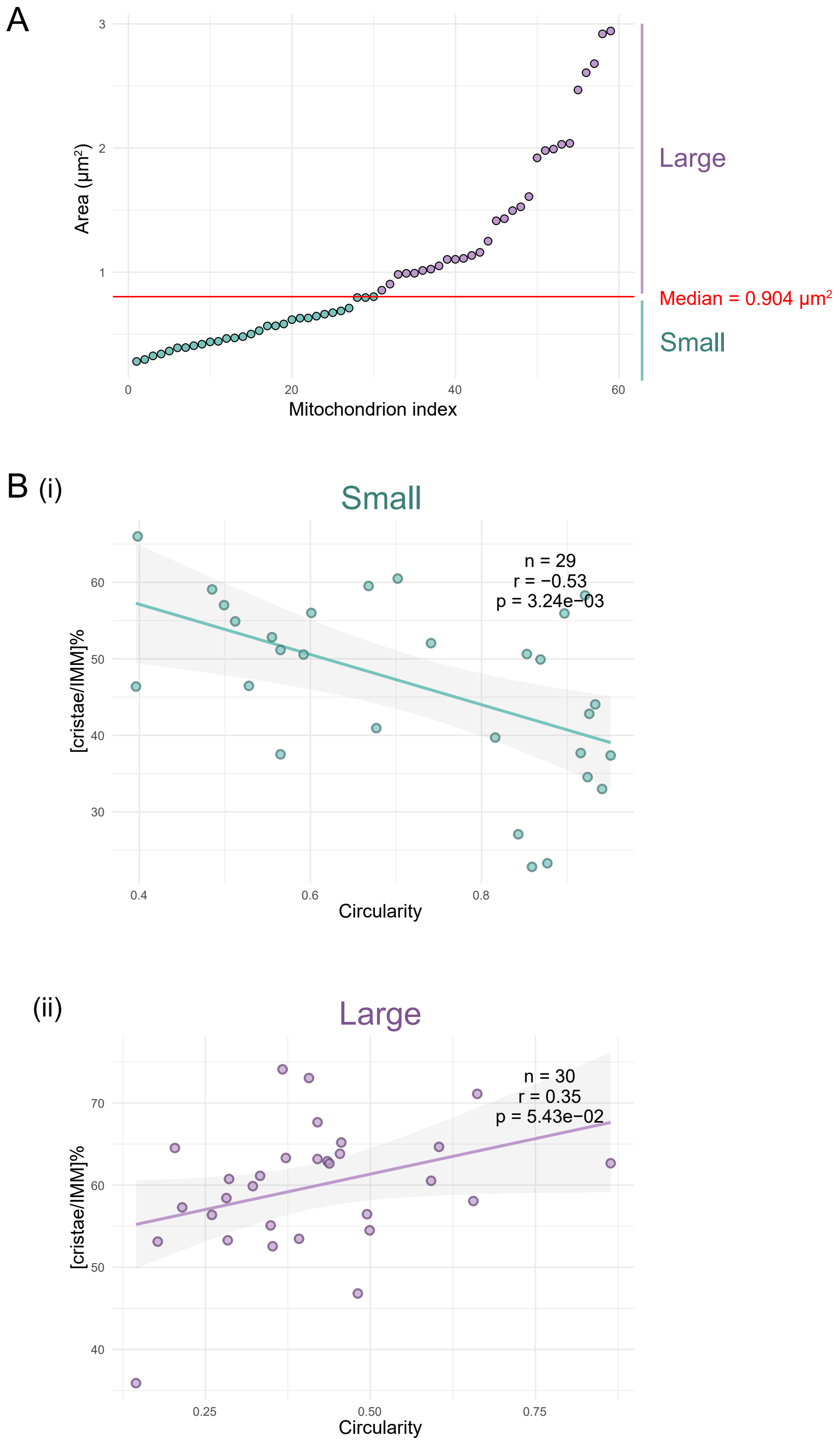

Journal of Cell Science • Supplementary information

Fig. S2. The relationship between [cristae/IMM]% and circularity in different size-groups of mitochondria. (A) Area values (μm^2) of mitochondria plotted in ascending order. The data was divided into two size-groups by the median value, indicated by the red line (median = 0.904 μm^2). (B) The percentage of cristae length out of the total IMM length in each mitochondrion ([cristae/IMM]%) was plotted against the circularity measured for the shape of (i) "small"-sized mitochondria and (ii) "large"-sized mitochondria. A circularity value of 1 indicates a perfect circle. A Pearson's correlation test (two-tailed) was performed. The line represents a linear regression fit and the shaded area indicates the 95% confidence interval. n, number of analyzed mitochondria; r, Pearson's correlation; p, P-value.

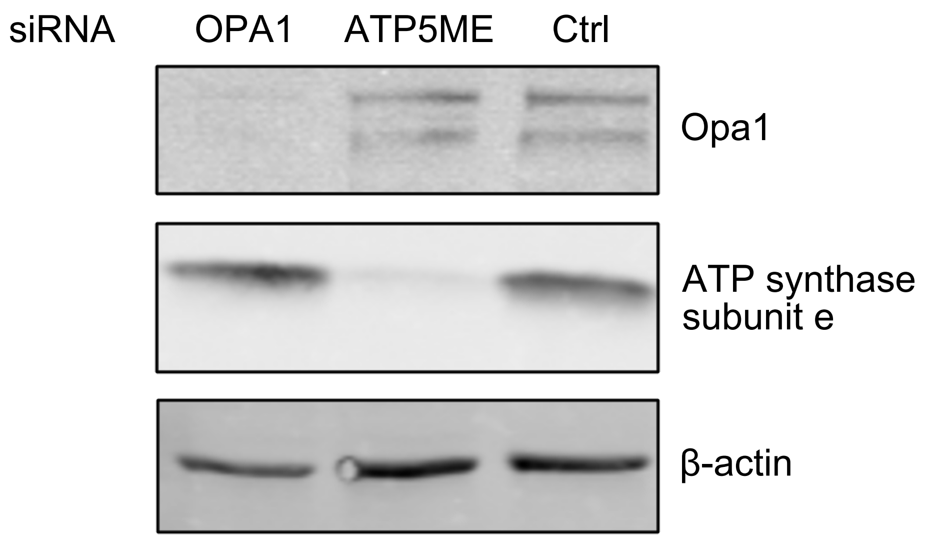

Fig. S3. Efficient knockdown of cristae shaping proteins. Western blots for validation of the siRNA pools used to knockdown OPA1 and ATP5ME. For transfection, 2×10^5 HeLa cells were seeded into a 60 mm culture dish with regular culture medium. Upon 72 h, cells were harvested and subjected to western blotting using the respective antibodies. β-actin served as loading control.

Journal of Cell Science • Supplementary information
